# Inhibition of SUMOylation enhances DNA hypomethylating drug efficacy to reduce outgrowth of hematopoietic malignancies

**DOI:** 10.1038/s41375-023-01838-8

**Published:** 2023-02-15

**Authors:** Jessie S. Kroonen, Ilona J. de Graaf, Sumit Kumar, Dennis F. G. Remst, Anne K. Wouters, Mirjam H. M. Heemskerk, Alfred C. O. Vertegaal

**Affiliations:** 1 Department of Chemical and Cell Biology, Leiden University Medical Centre, Leiden, The Netherlands; 2 Department of Hematology, Leiden University Medical Centre, Leiden, The Netherlands

**Keywords:** B-cell lymphoma, Cell signalling

## Abstract

Combination therapies targeting malignancies aim to increase treatment efficacy and reduce toxicity. Hypomethylating drug 5-Aza-2’-deoxycytidine (5-Aza-2’) enhances transcription of tumor suppressor genes and induces replication errors via entrapment of DNMT1, yielding DNA-protein crosslinks. Post-translational modification by SUMO plays major roles in the DNA damage response and is required for degradation of entrapped DNMT1. Here, we combine SUMOylation inhibitor TAK981 and DNA-hypomethylating agent 5-Aza-2’-deoxycytidine to improve treatment of MYC driven hematopoietic malignancies, since MYC overexpressing tumors are sensitive to SUMOylation inhibition. We studied the classical MYC driven malignancy Burkitt lymphoma, as well as diffuse large B-cell lymphoma (DLBCL) with and without MYC translocation. SUMO inhibition prolonged the entrapment of DNMT1 to DNA, resulting in DNA damage. An increase in DNA damage was observed in cells co-treated with TAK981 and 5-Aza-2’. Both drugs synergized to reduce cell proliferation in vitro in a B cell lymphoma cell panel, including Burkitt lymphoma and DLBCL. In vivo experiments combining TAK981 (25 mg/kg) and 5-Aza-2’ (2.5 mg/kg) showed a significant reduction in outgrowth of Burkitt lymphoma in an orthotopic xenograft model. Our results demonstrate the potential of tailored combination of drugs, based on insight in molecular mechanisms, to improve the efficacy of cancer therapies.

## Introduction

Combining complementary strategies to target cancer-inducing or tumor-sensitizing pathways is a cornerstone in cancer treatment [1]. Combining anti-cancer therapies enhances their efficacy, via synergy or additive efficacy, potentially reducing drug resistance. Single compound drug toxicity can be prevented by reducing drug dosing in combination treatment [2]. Thus, there is a need for novel combination therapies, combining existing treatments to increase their efficacy and reduce toxicity.

Epigenetic dysregulation is often linked to cancer, via amongst others altered transcription patterns of oncogenic and tumor suppressor genes [3]. Epigenetic regulation includes DNA methylation, modification of histones and chromatin remodeling, regulating expression and/or repression of the genome [4]. Some epigenetic alterations are involved in oncogenic transformation of cells [5] and the reversibility of epigenetic modifications makes them an interesting target for therapy.

DNA methyltransferases are in charge of DNA methylation site maintenance during cell division, in which they methylate CpG sites and consequently silence genes [6, 7]. Overexpression of DNA methyltransferases has been frequently found in human malignancies, potentially involved in silencing tumor suppressor pathways [8, 9]. Hypomethylating agents are broad re-programmers of DNA methylation and have been around for over 40 years [10]. 5-Aza-2’-deoxycytide (5-Aza-2’) is effective, however also resistance to therapy is common [11, 12]. 5-Aza-2’ has a dual role of action, in short hypomethylation and thus reactivation of tumor suppressor genes as described and inducing cytotoxic stress via DNA-protein crosslinks (DPC) due to the entrapment of DNMT1 to the DNA [13]. A recent study has revealed that the mechanism employed by cells to clear 5-Aza-2’ trapped DNMT1 from the chromatin, is dependent on SUMOylation [14, 15]. This led to our hypothesis that combining SUMOylation inhibitor TAK981 [16] with 5-Aza-2’ could yield an effective combination therapy based on insight in the molecular mechanisms employed by these drugs.

SUMOs (small ubiquitin-like modifiers) are post-translational modifications (PTMs) involved in e.g. regulation of cell cycle progression, DNA damage response and transcription [17–19]. SUMOs can be conjugated and removed from target proteins in a dynamic manner. SUMO conjugation enables protein-protein interactions, regulating protein localization, degradation or enzymatic activity [18]. In recent years, highly specific SUMOylation inhibiting small molecules have been developed for cancer therapy [16, 20, 21]. Small molecule ML792 and its analogue TAK981 specifically block the SUMO activating enzyme (SAE), consequently impairing the SUMOylation cascade thereby blocking target SUMOylation [16, 22]. The small molecule inhibitors form irreversible adducts with SUMO in an ATP dependent manner facilitated by the SAE itself. Inhibitor-SUMO adducts bind rigidly to SAE2/UBA2, the catalytic subunit of the SAE heterodimer, blocking the enzyme. The inhibitors are specific for the SUMO E1 enzyme and don’t block the ubiquitin E1 enzyme UAE [22, 23]. Specificity was furthermore confirmed against a panel of 366 different ATPases [22].

SUMO machinery components are regularly overexpressed in many different cancer tissues [24]. Cell cycle progression of cancer cells is dependent on SUMOylation [25]. Rapidly cycling MYC-driven tumors are highly sensitive towards SUMOylation inhibition [26, 27] and therefore a potential interesting target for SUMOylation inhibition. The classical MYC-driven cancer is Burkitt lymphoma [26, 27]. Furthermore approximately 15–30% of diffuse large B-cell lymphomas (DLBCL) has a *MYC* translocation, often in combination with partner mutations, which is a negative predictor of disease outcome [28, 29]. Therefore, we chose a panel of ten B cell lymphomas, including five Burkitt lymphoma cell lines and five DLBCL cell lines (three germinal centre B-cell (GBC) DLBCLs and two activated B-cell (ABC) DLBCLs), as a model system to test the novel combination therapy of TAK981 and 5-Aza-2’.

In this study we inhibit the two highly dynamic systems of chromatin methylation and protein SUMOylation to target B cell lymphoma tumor cell growth. The combination of TAK981 and 5-Aza-2’ led to a synergistic decrease in tumor cell growth in vitro and in vivo via induction of DNA damage, which could not be cleared due to the inhibition of SUMOylation.

## Results

### SUMOylation inhibition prevents 5-Aza-2’-deoxycytidine induced proteasomal degradation of DNMT1

**Fig. 1:**
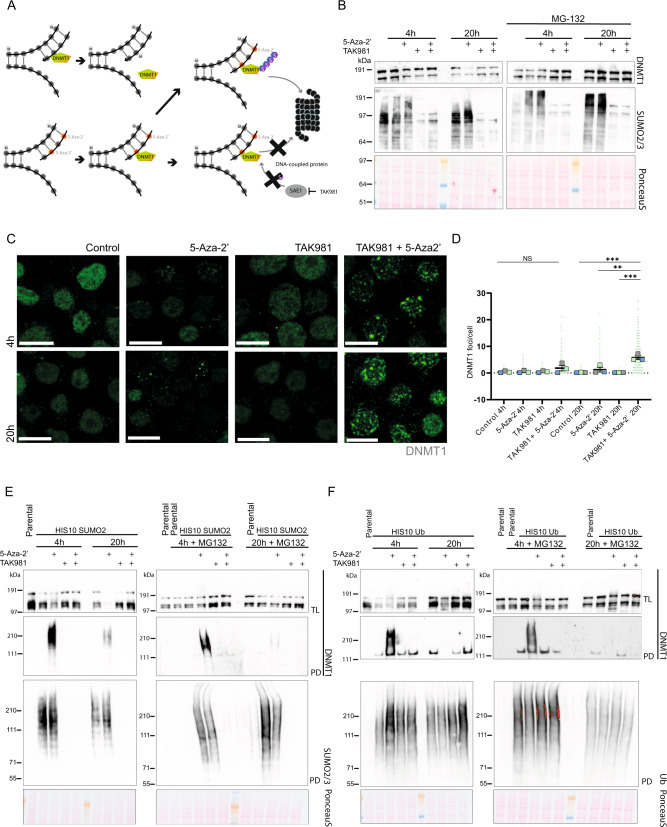
SUMOylation inhibition rescues DNMT1 degradation upon 5-Aza-2’ treatment and contributes to prolonged presence of DNMT1 in foci. **A** Mechanistic model for combining the drugs 5-Aza-2’ and TAK981. 5-Aza-2’ incorporates into the DNA at the site of cytidine. DNMT1 binding to 5-Aza-2’ gets trapped and is subsequently massively SUMOylated, ubiquitinated and degraded by the proteasome. Upon 5-Aza-2’and TAK981 treatment, DNMT1 remains entrapped at the DNA. **B** Namalwa cells were cultured in suspension and treated for 4 or 20 h with 1 µM 5-Aza-2’ and/or 1 µM TAK981 or DMSO 0.1% as control with or without MG132 10 µM for 4 h and 2.5 µM for 20 h. Total lysates were analyzed by immunoblotting using antibodies directed against DNMT1 and SUMO2/3. PonceauS staining was used as control. **C** DNMT1 foci were visualized by confocal microscopy. Namalwa cells were treated for 4 or 20 h with 1 µM 5-Aza-2’ and/or 1 µM TAK981 or DMSO 0.1% as control and cells were spun onto glass coverslips and stained. Representative images are depicted. Scale bars represent 10 µm. **D** Quantification of images from **C**. The graph depicts DNMT1 foci. Dots represent the numbers of DNMT1 foci/cell. 100 cells per replicate were analyzed (*n* = 3). *P-value* ** ≤ 0.01, *** ≤ 0.005. One-way ANOVA was performed with Graphpad Version 9.3.1. **E**, **F** Respectively show Ni-NTA pulldown of His10-SUMO2- and His10-ubiquitin. Namalwa cells were cultured in suspension and treated for 4 or 20 h with 1 µM 5-Aza-2’ and/or 1 µM TAK981 or DMSO 0.1% as control with or without MG132 10 µM for 4 h and 2.5 µM for 20 h. Total lysates (TL) and elutions from His10 pulldowns (PD) were analyzed by immunoblotting with antibodies directed against DNMT1, SUMO2/3 or ubiquitin. Equal loading was verified with Ponceau S staining.

We addressed the question whether 5-Aza-2’ and the SUMO E1 inhibitor TAK981 could be used to improve treatment of MYC-driven hematopoietic malignancies. As a model system, we used the Burkitt lymphoma cell line Namalwa. In addition, we used U2OS, a cell line commonly used to study the DNA damage response that is practical to use for microscopy. The molecular mechanism underlying the combination therapy is shown in Fig. 1A. 5-Aza-2’ traps the methyltransferase DNMT1 on DNA, leading to a block in replication and a reduction in methylation. Trapped DNMT1 is degraded by the proteasome in a SUMOylation dependent manner in HeLa cells [14].

To investigate synergy between these drugs in Burkitt lymphoma, we treated Namalwa with 5-Aza-2’ and/or inhibited SUMOylation (Fig. 1B). A reduction in DNMT1 levels was found 4 h after the start of the 5-Aza-2’ treatment and most of the DNMT1 was degraded at 20 h. DNMT1 degradation could be blocked efficiently by inhibition of SUMOylation by TAK981 as well as by blocking the proteasome using MG132. Thus, 5-Aza-2’ induces DNMT1 degradation in Namalwa in a SUMOylation and proteasome-dependent manner.

Next, we studied subcellular localization of DNMT1 in Namalwa. 5-Aza-2’ treatment induced bright nuclear DNMT1 foci. Combination of 5-Aza-2’ and TAK981 treatment significantly increased the amount of DNMT1 foci after 20 h (Fig. 1C, D). Increased numbers of these foci upon combination treatment can be explained by the blockage of SUMOylation with TAK981, consistent with a critical role for SUMOylation in the degradation of DNMT1. Similar results were obtained in U2OS cells (Supplementary Fig. 1C, D).

To study SUMOylation of DNMT1, we performed SUMO2 pulldown experiments in Namalwa and U2OS cells because SUMO2 is the most highly expressed SUMO family member in mammalian cells [30]. Namalwa and U2OS cells stably expressing His10-SUMO2 were treated with 5-Aza-2’ and/or SUMO inhibitor TAK981 for 4 h or 20 h in the presence and absence of the proteasome inhibitor MG132. Cells were lysed, His10-SUMO2 conjugates were purified and analyzed by immunoblotting. Our results demonstrate that 5-Aza-2’ treatment induced striking SUMOylation of DNMT1 as expected, and subsequent degradation over time (Fig. 1E and Supplementary Fig. 1A). SUMO inhibitor TAK981 completely prevented SUMOylation of DNMT1.

To study a role for ubiquitin in this process, these experiments were repeated, now using His10-ubiquitin instead of His10-SUMO2. His10-ubiquitin pulldown and subsequent analysis of DNMT1 showed to some extent similar degradation over time of DNMT1 as we have observed for the SUMOylated fraction of DNMT1 (Fig. 1F and Supplementary Fig. 1B). Interestingly, 5-Aza-2’-induced ubiquitination of DNMT1 at 4 h is completely lost upon blocking SUMOylation with TAK981, showing that DNMT1 SUMOylation is required for its ubiquitination. This can be explained by STUbL (SUMO targeted ubiquitin ligase) RNF4 dependent proteolysis of DPCs as described previously [15]. Taken together, our findings confirm that combining 5-Aza-2’ and TAK981 can efficiently be used to trap DNMT1 at the chromatin in Namalwa and U2OS.

### Proteomics analysis of SUMO2 targets upon 5-Aza-2’-deoxycytidine treatment

**Fig. 2:**
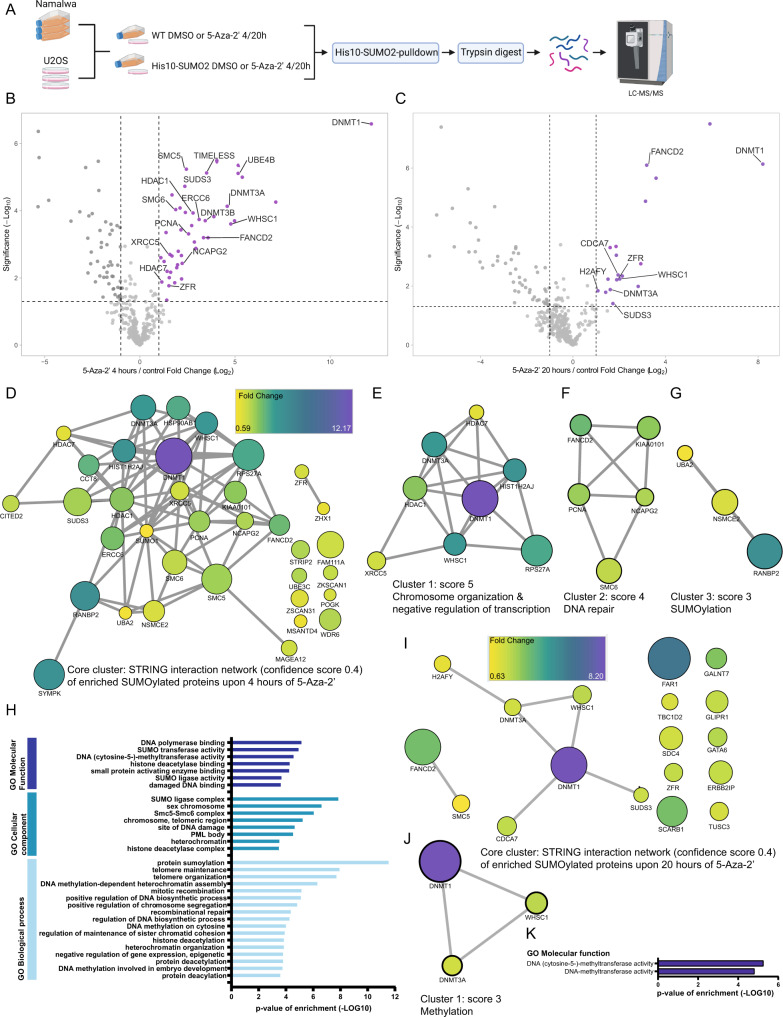
5-Aza-2’ treatment induces SUMOylation of DNA damage response factors and chromatin components in U2OS cells. **A** Experimental overview created with BioRender.com for the SUMO2 target identification upon 4 or 20 h of 5-Aza-2’ treatment in U2OS and Namalwa cells. U2OS and Namalwa cells stably expressed His10-SUMO2. His10-SUMO2 targets were enriched via Ni-NTA pulldown. Proteins were trypsin digested and prepared for LFQ mass spectrometry. Four replicates were prepared per condition and analyzed by nano flow LC-MS/MS. **B**, **C** respectively show volcano plots visualizing all identified SUMOylated proteins in U2OS His10-SUMO2 upon 4 or 20 h of 5-Aza-2’ treatment (1 µM) compared to control. His-SUMO2 target proteins were enriched via Ni-NTA pulldown, followed by trypsin digestion and LFQ mass spectrometry, peptides were identified by LC-MS/MS. Dashed lines represent cut off at a foldchange of two (log2 = 1) and *p-value* of 0.05 (-log10 = 1.3) (*n* = 4). **D** STRING network analysis of enriched SUMOylated proteins upon 4 h of 5-Aza-2’ treatment with Cytoscape Software at a confidence score of 0.4. The interaction network visualizes fold change via node color as indicated in the scale bar (fold change of: 0.59–12.17) and significance indicated with node size. **E** MCODE was used to extract the most interconnected clusters from the STRING network analysis in **D**. Cluster 1 represents proteins involved in chromosome organization and negative regulation of transcription. **F** Cluster 2 represents DNA damage response proteins. **G** Cluster 3 represents proteins involved in SUMOylation. **H** Bar-graph visualizes Gene Ontology enrichment analysis of proteins SUMOylated upon 4 h of 5-Aza-2’ treatment, for GO molecular functions, GO Cellular components and GO Biological processes compared against the reference humane proteome. Only pathways significantly enriched and with a fold enrichment of more than 20 are shown. **I** STRING network analysis of enriched SUMOylated proteins upon 20 h of 5-Aza-2’ treatment with Cytoscape software at a confidence score of 0.4. The interaction network visualizes fold change via node color as indicated in the scale bar (fold change of: 0.63–8.2) and significance indicated with node size. **J** MCODE was used to extract the most interconnected clusters from the STRING network analysis in **I**. Cluster 1 represents proteins involved in methylation. **K** Bar-graph visualizes Gene Ontology enrichment analysis of proteins SUMOylated upon 20 h of 5-Aza-2’ treatment, for GO molecular functions. Only pathways significantly enriched and with a fold change of more than 20 are shown. Source data are provided as Source data file_MS or _GeneOntology.

Subsequently, we set out to identify the SUMO2 target proteins that are responsive to 5-Aza-2’ treatment using an unbiased proteomics approach. His10-SUMO2 target proteins were enriched from Namalwa and U2OS treated with 5-Aza-2’ or DMSO for 4 h or 20 h (Fig. 2A) [31]. SUMO2 conjugated proteins were identified by mass spectrometry and quantified. As expected, DNMT1 is the most enriched SUMOylated protein upon 4 h and 20 h of 5-Aza-2’ treatment (Figs. 2B, C, S2A and B). Although DNMT1 can be found at both time points, the fold change drops considerably after 20 h of 5-Aza-2’ treatment, corroborating our pulldown experiments (Fig. 1E, F, Supplementary Fig. 1A, B). Furthermore, the methylases DNMT3A and DNMT3B as well as several DNA damage response proteins, including ERCC6, XRCC5, FANCD2, and SMC5/6 complex, were identified in our screen.

The majority of the proteins increased in SUMOylation upon 5-Aza-2’ treatment have functions related to chromatin biology. STRING network analysis (Fig. 2D, I) followed by MCODE sub-clustering showed specific networks linked to chromosome organization, negative regulation of transcription (Fig. 2E), DNA repair (Fig. 2F) and SUMOylation (Fig. 2G) for proteins identified upon 4 h of 5-Aza-2’ treatment and methylation for proteins identified after 20 h of 5-Aza-2’ treatment (Fig. 2J). Gene Ontology (GO) analysis was performed for 5-Aza-2’ induced SUMOylated proteins after 4 and 20 h of treatment, including GO biological processes, GO cellular components and GO molecular function. GO analysis confirmed that identified proteins were enriched for processes involved in chromatin-related processes; DNA repair, chromosome maintenance and epigenetic regulation (Fig. 2H, K). Our data highlight DNA methylases and DNA damage response factors as 5-Aza-2’ responsive SUMO2 targets.

### Entrapment of DNMT1 by 5-Aza-2’-deoxycytidine combined with SUMOylation inhibition increases DNA damage

**Fig. 3:**
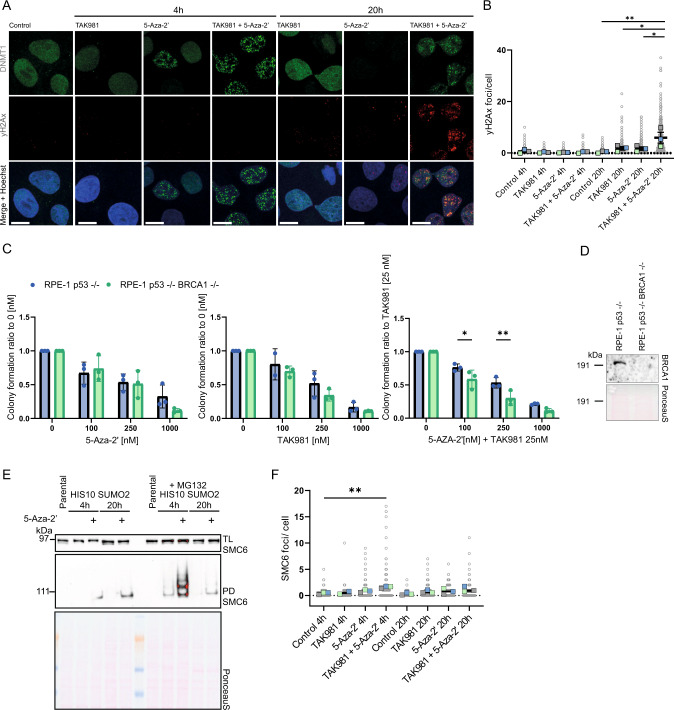
The combination treatment of 5-Aza-2’ and TAK981 induces DNA damage. **A** γH2Ax and DNMT1 foci visualized with confocal microscopy. U2OS cells cultured on glass cover slips were treated with 1 µM 5-Aza-2’ and/or 1 µM TAK981 or DMSO 0.1% as control for 4 or 20 h. Slides were stained with DNMT1 and γH2Ax antibodies. The panel shows representative images of each condition, single DNMT1 stain, single γH2Ax stain and a merged image including Hoechst staining. Scale bar represents 25 µm **B** γH2Ax foci quantification of images from **A**. Dots represent numbers of γH2Ax foci/cell using ~100 cells per replicate (*n* = 3). *P-value* * ≤ 0.05. One-way ANOVA was used, followed by Tukey’s multiple comparisons with Graphpad prism Version 9.3.1. **C** BRCA1 knock out in RPE-1 p53^-/-^ cells was validated via immunoblotting. PonceauS staining was used as loading control. **D** Colony formation analysis of homologous recombination (HR) deficient cell line RPE-1 p53^-/-^ BRCA1^-/-^ vs RPE-1 p53^-/-^ 2,500 cells were seeded per well in 6-well plates, treated with 5-Aza-2’ and/or TAK981 at the indicated doses and grown for 14 days. Subsequently, cells were fixed, stained with crystal violet and quantified. *P-value* * ≤ 0.05, ** ≤ 0.01. Two-sided *t*-test RPE-1 p53^-/-^ BRCA1^-/-^ vs RPE-1 p53^-/-^ per treatment condition with Graphpad Version 9.3.1. **E** Ni-NTA pulldown of His10-SUMO2- for validation of targets from mass spectrometry analysis (Fig. 2). U2OS cells were cultured in suspension and treated for 4 or 20 h with 1 µM 5-Aza-2’ or DMSO 0.1% control with or without MG132 10 µM for 4 h and 2.5 µM for 20 h. Total lysate input and His10-SUMO2- pulldown elutions were analyzed by immunoblotting using SMC6 antibody. Equal loading was verified with Ponceau S staining. **F** SMC6 foci were identified via confocal microscopy upon treatment with 1 µM 5-Aza-2’ and/or 1 µM TAK981 for 4 or 20 h and quantified. Dots represent numbers of SMC6 foci/cell using ~50–100 cells per replicate (*n* = 3). *P-value* * ≤ 0.05, ** ≤ 0.01. One-way ANOVA, Tukey’s multiple comparisons with Graphpad prism Version 9.3.1.

Based on our SUMO2 proteomics results and the drug mode of action, we set out to study induction of DNA damage by 5-Aza-2’ treatment and SUMOylation inhibition. U2OS cells were treated with 5-Aza-2’ to trap DNMT1 to DNA and/or SUMO E1 inhibitor TAK981 for 4 h or 20 h to block clearance of the DNMT1 DPCs. Cells were fixed and immunostained for DNMT1 and DNA double strand break marker γH2Ax. Single treatment with TAK981 and 5-Aza-2’ for 20 h led to modest increases in γH2Ax foci (Fig. 3A, B). Strikingly, γH2Ax foci were strongly increased upon combination treatment with 5-Aza-2’ and TAK981 for 20 h (Fig. 3A, B).

Next, we investigated the sensitivity of homologous recombination deficient cells (RPE-1 p53-/- BRCA1 -/-) (Fig. 3D) for 5-Aza-2’ and SUMO E1 inhibitor TAK981 treatment. Cells were seeded at low density, treated with the indicated drug concentrations and tested for drug sensitivity in a colony formation assay (Fig. 3C). We observed a significant reduction in colony formation for homologous recombination deficient cells in response to combination treatment compared to wild-type cells, indicating that homologous recombination is required for repair of the induced DNA double strand breaks. Furthermore, we identified SMC6 by proteomics as SUMO2 target responsive to 5-Aza-2’ treatment (Fig. 2B and Supplementary Fig. 2A, B) and confirmed increased SUMOylation of SMC6 in response to 5-Aza-2’ by immunoblotting (Fig. 3E). Consistently, we observed an increase in SMC6 foci 4 h post treatment with TAK981 and 5-Aza-2’ (Fig. 3F). Taken together our data show striking induction of DNA damage in response to 5-Aza-2’ and TAK981 combination treatment and the requirement for the homologous recombination pathway for resolution of the induced DNA damage.

### SUMOylation inhibition enhances 5-Aza-2’-deoxycytidine efficacy in a hematopoietic cell panel

To evaluate the 5-Aza-‘2 and TAK981 combination treatment in B cell lymphoma, we set up a panel of ten different B cell lymphoma cell lines (Table 1), including Burkitt lymphomas which are classically MYC-driven tumors and therefore of interest for treatment with TAK981 [26, 27]. In addition, DLBCLs were added of which approximately 15–30% have a *MYC* translocation, which together with *BCL2* and/or *BCL6* translocation or amplification are known as ‘double’ or ‘triple’-hit lymphomas. DLBCLs with *MYC* translocation are more difficult to treat and therefore of interest to target with our combination therapy [28, 29].

**Table 1:** B cell lymphoma cell panel cell lines, specifics and mutational information.

Cell line	Specifics	*MYC* translocation	BCL2 or 6 translocation	p53 status	Ref.
Namalwa	Burkitt Lymphoma (EBV)	+	-	Mutant	[44]
RAMOS	Burkitt Lymphoma	+	-	Mutant	[45]
CA46	Burkitt Lymphoma	+	-	Mutant	[46]
DG75	Burkitt Lymphoma	+	-	Mutant	[47]
ST486	Burkitt Lymphoma (EBV)	+	-	Mutant	[46]
OCI-Ly3	Diffuse Large B-cell Lymphoma (ABC)	-	BCL2 (CNG)	WT	[48, 49]
U2932	Diffuse Large B-cell Lymphoma (ABC)	-	BCL2, BCL6	Mutant	[50]
SC-1	Diffuse Large B-cell Lymphoma (GCB)	+	BCL2	Mutant	[48, 51]
SUDHL4	Diffuse Large B-cell Lymphoma (GCB)	+	BCL2, BCL6	Mutant	[48, 52, 53]
SUDHL5	Diffuse Large B-cell Lymphoma (GCB)	+	BCL2	WT	[52, 53]

**Fig. 4:**
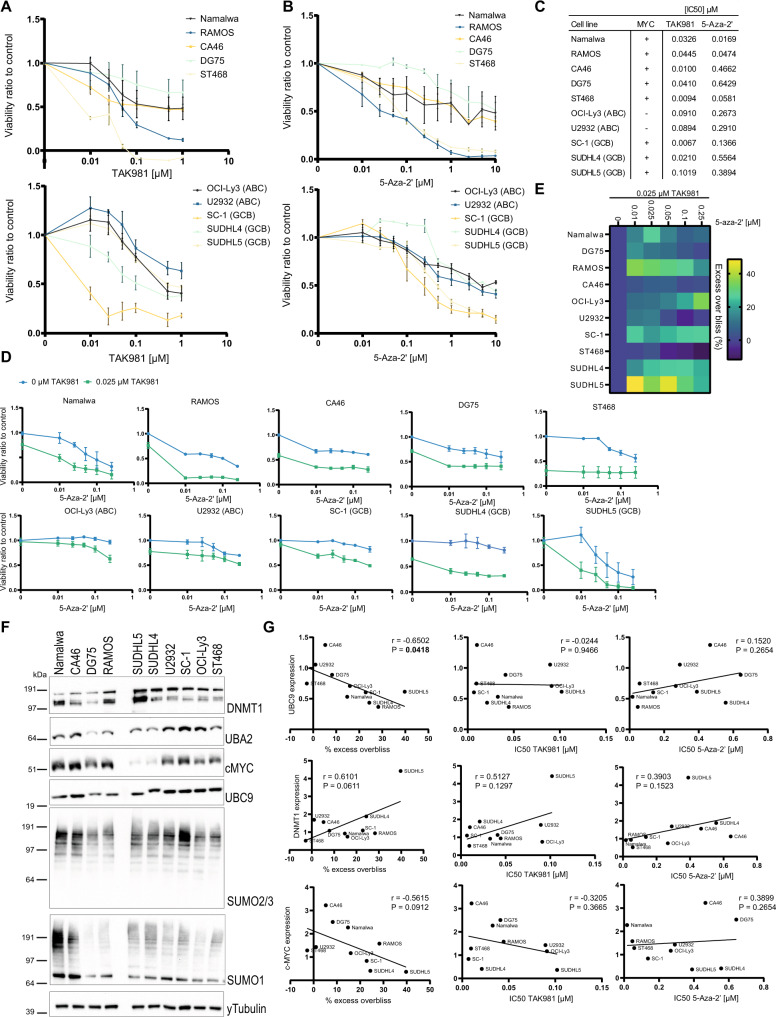
5-Aza-2’ and TAK981 synergize to reduce lymphoma tumor cell growth. **A** A panel of ten lymphoma cell lines was treated with TAK981 at a dose range of 0.01–1 µM and cell viability was measured after 4 days. Cells were divided over two graphs respectively: Burkitt Lymphoma cell lines and DLBCL cell lines. The graphs show cell viability in ratio to control per cell line. **B** The panel of ten lymphoma cell lines was treated with 5-Aza-2’ at a dose range of 0.01–10 µM and cell viability was measured after 4 days. Cells were divided over two graphs respectively, representing Burkitt Lymphoma cell lines or DLBCL cell lines. The graphs show cell viability in ratio to control per cell line. **C** IC50 values of the cell line panel for TAK981 0.00001–1 µM dose response and 5-Aza-2’ dose response 0.01–10 µM. IC50 values were calculated in Graphad Version 9.3.1. and *MYC* status for all cell lines ‘+’ indicates translocation of MYC gene (Table 1), ‘-‘ represents no change in *MYC* gene (Table 1). **D** The panel of ten lymphoma cell lines was treated with 5-Aza-2’ at the indicated dose range with or without 25 nM TAK981. Viability dose response curves were plotted individually per cell line. **E** Excess overbliss (%) of plots in **D** was calculated as detailed in methods section and visualized in a heat map (Higher % excess overbliss represents more synergy) for each dose of 5-Aza-2’ versus the same dose with 25 nM TAK981. **F** Total lysates of the panel of ten lymphoma cell lines were analyzed for protein expression levels of DNMT1, UBA2, MYC, UBC9, SUMO2/3 and SUMO1 by immunoblotting. γTubulin staining was used as a control. Immunoblotting of representative image of a total of *n* = 3 is shown. **G** Correlation of IC50 value and average excess overbliss percentage per cell line vs protein expression of DNMT1, UBC9 and MYC. Correlation was calculated in Graphpad Prism 9.3.1. Pearson r and *P-value*. Correlation data of the remainder of the proteins is depicted in Supplementary Fig. 2.

**Fig. 5:**
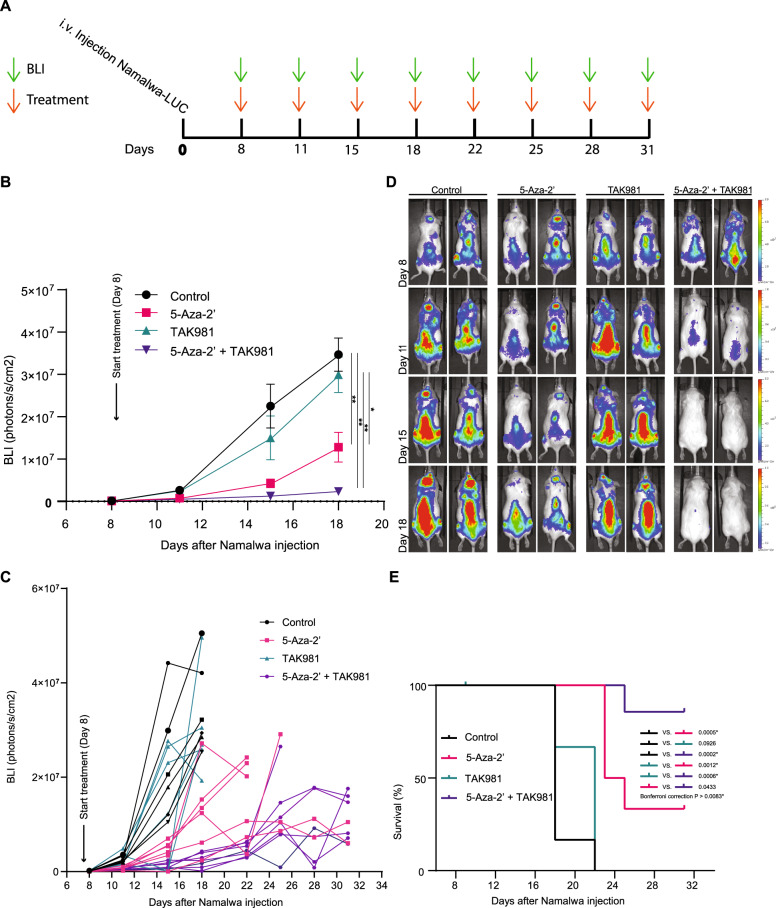
Efficient reduction in tumor cell outgrowth in vivo by combining 5-Aza-2’ and TAK981. **A** Graphic visualization of in vivo experimental time line. First bioluminescence (BLI) measurement was carried out on day 8, followed by subsequent rounds of treatment and BLI measurements two times per week. **B** NSG mice were transplanted with 1 × 10^5^ Namalwa-LUC cells via intra venous injection. Average BLI signal per group (*n* = 7) for the times when all mice were present in the experiment. Mice were treated with 5-Aza-2’ at 2.5 mg/kg and/or TAK981 at 25 mg/kg, control mice were treated with solvent HPBCD. Treatment occurred bi-weekly and was given intra-peritoneal. Two-way ANOVA analysis followed by Tukey multiple comparisons with Graphpad prism Version 9.3.1. *P-value* * ≤ 0.05, ** ≤ 0.01. **C** Data of **B** visualized per individual mouse over the time course when each mouse was present in the experiment. **D** Panel displays representative images of 2 mice per group for days shown in **B. E** Survival plot depicts survival of mice per group over the time course of the experiment.

All cell lines within our panel were sensitive towards SUMOylation inhibition (Fig. 4A) and 5-Aza-2’ treatment (Fig. 4B) in a dose dependent manner. IC50 calculations showed differences in the sensitivity between the cell lines towards both compounds (Fig. 4C). However, sensitivity towards single compounds was independent of *MYC* translocation status or DLBCL subtype (Supplementary Fig. 3D, E). Combination dosing included 5-Aza-2’ dose escalation (low dose range values of Fig. 4B) in combination with 25 nM of TAK981 (Fig. 4D). Via excess overbliss calculations [32], the percentage of synergy was studied for each cell line, demonstrating drug synergy in eight out of ten cell lines except for CA46 and ST468 cells (Fig. 4E). Remarkably, drug synergy was highest in the SUDHL5 cell line, which had the lowest level of MYC expression (Fig. 4F), however known to have *MYC* translocation (Table 1). Our results show strong potential of the drug combination for treatment of Burkitt lymphoma and DLBCL, even in a MYC-independent manner.

To investigate proteins that can serve as predictive biomarkers for successful combination treatment, we evaluated protein expression levels of candidate biomarkers including DNMT1, MYC, SUMO E1 enzyme UBA2 and SUMO E2 enzyme UBC9 as well as conjugation levels of SUMO1 and SUMO2/3 by immunoblotting (Fig. 4F). Next, we studied the correlation of the protein levels to single or combined drug sensitivity (Fig. 4F, G, Supplementary Fig. 3G). Interestingly, the SUMO E2 enzyme UBC9 significantly correlated with sensitivity towards the combination therapy, but not to single drug sensitivity. Also, DNMT1 expression is a potential biomarker for efficacy of combination therapy (Fig. 4F, G). Mutational status of tumor suppressor gene p53 is variable within our panel of B cell lymphoma cell lines (Table 1), however p53 status is not a distinctive marker for treatment efficacy (Supplementary Fig. 3F).

MYC expression did not correlate with sensitivity towards either 5-Aza-2’ or TAK981 treatment between cell lines within our panel (Fig. 4F, G, Supplementary Fig. 3D). However, upon comparison of DLBCLs with or without *MYC* translocation, DLBCLs positive for *MYC* translocation were more sensitive towards combination treatment (Supplementary Fig. 3E). Furthermore, we showed that inducible knock down of *MYC* in P493-6 cells desensitized this cell line towards SUMOylation inhibition (Supplementary Fig. 2A, B, C). Expression levels of MYC did not always correlate with *MYC* translocation status (Table 1 and Fig. 4F), potentially explaining the lack of correlation between sensitivity and MYC translocation status (Fig. 4G). In summary, 5-Aza-2’ and TAK981 have potential to synergistically inhibit tumor cell growth and UBC9 and DNMT1 expression could be potential biomarkers for sensitivity of lymphomas for the combination therapy.

### 5-Aza-2’-deoxycytidine efficacy is enhanced by SUMOylation inhibition in vivo *in* an orthotopic xenograft lymphoma model

Subsequently, we tested the 5-Aza-2’ and TAK981 combination therapy in an orthotopic xenograft Burkitt lymphoma model. The human Burkitt lymphoma cell line Namalwa was used as in vivo model. This cell line was an average responder to the in vitro combination therapy in our cell panel. Namalwa cells expressing luciferase were transplanted into immune deficient mice. The luciferase allowed us to track the cells in vivo upon luciferin injection with help of the In Vivo Imaging System (IVIS). 1 × 10^5^ Namalwa cells were injected intravenously, and engrafted for 7 days. Bioluminescence (BLI) was measured to visualize tumor growth. Mice were treated with 5-Aza-2’ and/or TAK981, which continued bi-weekly for 31 days after tumor engraftment (Fig. 5A). Treatment with TAK981 did not significantly reduce tumor cell growth in vivo, indicating that inhibition of SUMOylation is not sufficient to reduce tumor growth. In contrast, treatment with 5-Aza-2’ did significantly reduce tumor cell growth in vivo. Strikingly, the combination of 5-Aza-2’ and TAK981 strongly reduced tumor cell growth and increased survival (Fig. 5B, C, D, E). The combination therapy was well tolerated with no signs of toxicity (Supplementary Fig. 4). These data demonstrate the in vivo potential for TAK981 to enhance hypomethylation drug 5-Aza-2’ efficacy.

## Discussion

In this study we demonstrate the potential of combining hypomethylating drug 5-Aza-2’-deoxycytidine and SUMOylation inhibitor TAK981 for cancer therapy. By combining these drugs, we employed the synergistic effects of these two compounds. 5-Aza-2’ trapped DNMT1 to DNA and SUMOylation inhibition prevented clearing of trapped DNMT1, leading to induction of DNA damage and consequently impaired cell survival. In a panel of ten B cell lymphoma cell lines, including five Burkitt lymphomas and five DLBCLs, in vitro synergism of 5-Aza-2’ and SUMOylation inhibition was validated. In a Namalwa orthotopic xenograft model, significant in vivo tumor outgrowth reduction was obtained with 5-Aza-2’ and TAK981 combination therapy. Our results demonstrate that this drug combination is effective to reduce lymphoma outgrowth.

We evaluated the role of MYC overexpression by knocking down *MYC* expression in P493-6 Burkitt lymphoma cells. We observed decreased sensitivity towards TAK981 treatment upon MYC knockdown in agreement with literature [26, 27]. It should be noted that MYC knock down halts proliferation, which is known to de-sensitize cells for cytostatic therapies. Translocation status of MYC in our panel of ten lymphoma cell lines did not predict sensitivity. In addition, our in vivo experiments showed no significant reduction in tumor outgrowth in response to SUMOylation inhibition as single compound therapy, whereas literature suggest that SUMO abolishment by genetic knock down of the E1 enzyme does reduce tumor outgrowth in MYC dependent tumors [26, 27]. A potential explanation for this discrepancy is that TAK981 treatment does not lead to a continuous abolishment of SUMOylation in mice, because the compound is cleared by the mice in between treatments [16]. Our results highlight the need for developing combination therapies that are more efficient than single compound therapies.

Interestingly, expression of SUMO E2 enzyme UBC9 significantly correlated to drug synergy, indicating that low expression of UBC9 sensitized the cell lines in our panel for the combination therapy. Interestingly, DNMT1 expression shows an inversed correlative trend. In addition to UBC9, no other members of the SUMOylation cascade evaluated, showed a clear correlation with drug sensitivity.

Using an unbiased proteomics approach, we identified SUMO2 target proteins induced by 5-Aza-2’ treatment. The identified proteins are commonly related to chromatin regulation, genome maintenance and repair. DNMT1 is the main SUMO2 target induced by 5-Aza-2’ treatment as expected. In addition, SUMOylation of the methylases DNMT3A and -B and several DNA damage response proteins, including ERCC6, XRCC5, FANCD2 as well as the SMC5/6 complex were also induced by 5-Aza-2’ treatment. The SMC5/6 complex is localized to double-stranded breaks (DSBs) and promotes repair via homologous recombination (HR). SMC5/6 complex subunit Nse2 is a SUMO-ligase and SUMOylates amongst others the Scc1 subunit of cohesin to promote sister chromatid recombination (Palecek, 2018; Stephan et al., 2011). Upon replication fork stress, SMC5/6 SUMOylation and Nse2 SUMO-ligase activity are involved in the resolution of blockages preventing replication [33, 34]. Consistently, we found that cells develop more SMC6 foci upon combination treatment (Fig. 3F). Furthermore, cells deficient for homologous recombination (HR) are more sensitive towards 5-Aza-2’ treatment combined with TAK981. With the knowledge that proper functioning of the Structural Maintenance of Chromosome complex is at least partly dependent on SUMOylation, we hypothesize that this pathway is compromised by the 5-Aza-2’ TAK981 combination therapy. Combined, our data suggests that cells compromised in homologous recombination are more sensitive to DNA damage induced by 5-Aza-2’ and TAK981. BRCA1 and BRCA2 mutations responsible for the majority of homologous recombination deficient (HRD) cancers are prevalent in solid tumors of the breast, ovaries and prostate [35]. Therefore, it would be interesting to test whether these types of HRD cancers are also sensitive to the combination therapy of TAK981 and 5-Aza-2’.

Whether TAK981 synergizes with other DNA damaging agents to yield effective combination therapies remains to be investigated. Moreover, whether the combination therapy of TAK981 and 5-Aza-2’ is more effective, compared to current therapies that also cause DNA damage such as cyclophosphamide, doxorubicin or radiation or compared to other epigenetic therapies remains to be investigated as well. It should be noted that SUMOylation appears crucial for different DNA repair pathways [36]. All DNA damage inducing therapies are toxic, which is part of their effectiveness, and this therapy will not be an exception. Nevertheless, an important advantage of the synergy observed between TAK981 and 5-Aza-2’ is that lower doses of both drugs could be employed, limiting toxicity. Furthermore, the TAK981 and 5-Aza-2’ combination therapy could potentially prevent drug resistance or help to overcome resistance to other drugs.

Recently, other combination therapies with SUMOylation inhibitor TAK981 have been explored. Multiple myeloma is sensitive to combination therapy of TAK981 with lenalidomide or dexamethasone [37, 38]. Furthermore, Phase I and II clinical studies are ongoing with TAK981 in combination with several monoclonal antibody therapies including, Mezagitamab, Daratumumab, Rituximab and Pembrolizumab (NCT04776018, NCT04074330, NCT04074330, NCT0407433 (https://clinicaltrials.gov/ct2/results?cond=&term=TAK-981&cntry=&state=&city=&dist=)). Interestingly, another PTM drug inhibiting NEDD8 conjugation (Neddylation) has been combined with 5-Aza-2’ previously and showed synergism in pre-clinical and clinical AML experiments [39, 40]. In summary, we propose SUMOylation inhibitor TAK981 in combination with hypomethylation agent 5-Aza-2’ as a tailored drug combination for lymphoma, based on insight in molecular mechanisms.

## Materials and methods

### Compounds

TAK981 (Chemietek, Indianapolis, IN, USA) was dissolved in DMSO for in vitro usage and in 20% (2-Hydroxypropyl)-β-cyclodextrin (HPBCD, Merck, Darmstadt, Germany) for in vivo purposes. 5-Aza-2’-deoxycytidine (5-Aza-2’, Merck) was dissolved in DMSO for in vitro usage and in 20% HPBCD for in vivo purposes. MG132 (Merck) was dissolved in DMSO for in vitro experiments.

### Cell culture and cell lines

Cell lines used for the lymphoma panel; Burkitt lymphomas: Namalwa (Dept. of Hematology LUMC), CA46, RAMOS and DG75, ST468. Diffuse Large B-cell Lymphomas (DLBCL): SUDHL4, SUDHL5, OCI-Ly3, U2932, SC-1 (cell lines were obtained from Prof. Van den Berg, UMCG, Groningen). Namalwa was cultured in IMDM medium (Gibco™, Thermo Fisher Scientific, Waltham, MA, USA) supplemented with 10% Fetal Bovine Serum (FBS, South America Origin, Biowest) and 5% Penicillin-Streptomycin (P/S, Gibco™). The other cell lines were cultured in RPMI medium (Gibco™) supplemented with 5% P/S and 10% (CA46, RAMOS, DG75, SUDHL5, U2932 and Sc-1) or 20% (SUDHL4, OCI-Ly3, ST468) FBS. Cells were cultured in a humidified incubator at 37 °C and 5% CO_2_.

P493-6 Burkitt lymphoma cells (Obtained from Prof. Van den Berg, UMCG, Groningen [41]) with doxycycline (0.1 µg/mL) inducible *MYC* knock-down were cultured in RPMI medium (Gibco™) supplemented with 5% P/S and 10% FBS in a humidified incubator at 37 °C and 5% CO_2_.

U2OS-WT (ATCC® HTB-96™), U2OS His10-SUMO2 transduced (GFP-sorted), U2OS His10-ubiquitin (puromycin selected, 1 µg/mL) were cultured in DMEM (Gibco™) supplemented with 10% FBS and 5% P/S and cultured in a humidified incubator at 37 °C and 5% CO_2_.

RPE-1 p53^-/-^ and RPE-1 p53^-/-^ BRCA1^-/-^ (Noordermeer et al., 2018) were cultured in DMEM (Gibco™) supplemented with 10% FBS and 5% P/S and cultured in a humidified incubator at 37 °C and 5% CO_2_.

Namalwa cells grown for proteomics analysis were cultured in roller bottles (VWR, Radnor, PA, USA). Roller-bottles maximally held 1 L of culture at an optimal density between 0.25 × 10^6^–1.5 × 10^6^ cells/mL in a closed system (no gas-exchange) in a 37 °C temperature regulated room with roller-system.

All cell lines were regularly tested for mycoplasma contamination and identity was confirmed by STR profiling.

### Western blotting

Total cell lysates of Namalwa cells treated with TAK981 (1 µM) and/or 5-Aza-2’ (1 µM) or DMSO 0.01% were analyzed by western blotting. MG132 (10 µM for 20 h of treatment and 2.5 µM for 4 h of treatment) was used for proteasome inhibition conditions. Total lysates were prepared on ice in SNTBS buffer (2% SDS, 1% NP40, 50 mM Tris pH 7.5, 150 mM NaCl) followed by incubation for 10 min at 100˚C. Size separation of proteins was performed on precast 4–12% Bis-Tris gradient gels (Thermo Fisher Scientific) for total lysates or 3–8% Tris-Acetate (Thermo Fisher Scientific) for pulldown elution samples (described below in methods section His10-SUMO/Ub pulldown). Size-separated proteins were transferred to nitrocellulose membranes (0.45 µm, Amersham Protran Premium (Merck)). Primary antibodies against DNMT1 (rabbit, 1:1000, 5032 S, Cell Signaling Technology, Leiden, NL), SUMO2/3 (1:500, mouse monoclonal 8A2, University of Iowa), ubiquitin (1:5000, sc8017, Santa Cruz, Dallas, TX, USA) were incubated with membrane in 5% milk powder in PBS − 0.05% Tween20. Goat anti-mouse IgG-HRP (1:2500) and Donkey anti-rabbit IgG-HRP (1:10 000) were used as secondary antibodies in 5% milk. Signal was detected using Pierce ECL2 (Life Technologies, Carlsbad, CA, USA) and imaged using the iBright CL1500 (Invitrogen™ iBright Imaging Systems, Thermo Fisher Scientific).

### Microscopy immunostaining

U2OS and Namalwa cells were seeded or spun onto glass coverslips, respectively at 50,000 cells per coverslip. Following treatment with TAK981 (1 µM) and/or 5-Aza-2’ (1 µM), or control DMSO (0.01%), cells were fixed with 4% paraformaldehyde for 15 min, followed by washing 3 times with PBS. Cells were permeabilized with 1% Triton X-100 in PBS for 15 min at room temperature, subsequently blocked in 0.1 M Tris-HCL pH 7.5, 0.15 M NaCl, 5 mg/ml Boehringer Blocking Reagent (TNB) for 15 min at room temperature and incubated with primary antibodies DNMT1 (1:500, ab13537, Abcam, Cambridge, United Kingdom), γH2Ax (1:500, 9718 S, Cell Signaling Technology, Leiden, NL), SMC6 (1:500, A300-237A, Bethyl Laboratories, Waltham, MA) diluted in TNB for 1 h. Cells were washed three times in PBS −0.05% Tween20 and incubated with secondary antibodies 1:500 (anti-mouse conjugated Alexa-488, anti-rabbit conjugated Alexa-568) for 1 h. Glass coverslips were washed three times with PBS −0.05% Tween20, followed by PBS −0.05% Tween20 with 10 mg/mL Hoechst 33342 (Merck) for DNA staining incubated for 20 min. Subsequently coverslips were dehydrated and mounted onto glass slides using ProLong™ Gold Antifade Mountant (Merck).

### Confocal microscopy and image analysis

Imaging was performed with use of Leica SP8 confocal microscopy. Images were acquired with the 64x objective (oil) for U2OS cells and 100x objective (oil) for Namalwa cells. For imaging, frames of 1024 × 1024 pixels were used, z-stacks of 15 steps with a total size of 7.5 µm were obtained for all images. For each sample within every replicate, three individual fields were imaged, laser power was fixed for all samples within each replicate and adjusted to prevent overexposure. ImageJ (v1.53f51) was used to analyze the images and the BIC Macro Toolkit by Universität Konstanz Bioimaging Centre for foci quantification. In brief, maximum projections were generated from z-stack images, nuclear areas were selected based on Hoechst staining. Foci were identified based on the Find Maxima function, indicating a fixed noise ratio for every individual antibody set. Subsequently, foci were counted within the Macro.

Graphpad prism version 9.3.1 was used to calculate differences in DNMT1, γH2Ax and SMC6 foci between different treatments for each time point. One-way ANOVA was followed by Tukey multiple comparisons to calculate differences between every treatment alpha of 0.05 was considered significant.

### His10-SUMO/ubiquitin purification

Proteins conjugated to His10-SUMO2 or His10-ubiquitin were purified as described previously [31]. U2OS and Namalwa cells stably expressing His10-SUMO2 or His10-ubiquitin were lysed in 6 M Guanidine-HCL, 100 mM Sodium phosphate, 10 mM Tris, buffered at a pH of 7.8 and subsequently snap frozen. Lysates were thawed at room temperature, sonicated 2x for 10 s, supplemented with 5 mM β-mercaptoethanol and 50 mM imidazole pH 8.0. Samples were equalized using the bicinchoninic acid (BCA) Protein Assay (Merck). Ni-NTA beads (30210, Qiagen, Hilden, Germany) were added to the lysates and incubated overnight at 4 ˚C. Ni-NTA beads were washed extensively. Purified proteins were eluted three times in one bead volume of 7 M urea, 100 mM sodium phosphate, 10 mM Tris pH 7.0, and 500 mM imidazole pH 7.0. Elutions obtained for immunoblot analysis were supplemented with LDS sample buffer. Elutions for mass spectrometry analysis were trypsin digested.

### Mass Spectrometry

#### In solution digestion and stage tipping

His10-SUMO2 purified samples were concentrated through 100 kDa cutoff filters and supplemented with 50 mM ammonium bicarbonate (ABC). Subsequently, samples were reduced with 1 mM Dithiothreitol (DTT), alkylated using 5 mM chloroacetamide and reduced again with 6 mM DTT. Urea was diluted to 2 M with 50 mM ABC for trypsin (V5111, Promega, Madison, WI, USA) digestion in a ratio of 1:100 enzyme-to-protein overnight and light protected at room temperature. After digestion, peptides were acidified with 2% trifluoroacetic acid (TFA) and then desalted and concentrated on triple-disc C18 reverse phase StageTips (Rappsilber et al., 2007). Peptides were eluted with 33.3% acetonitrile (ACN), vacuum dried and dissolved in 0.1% folic acid.

#### LC-MS/MS analysis and data processing

Peptides were analyzed by nanoflow liquid C18 chromatography using an Ultimate 3000 nano HPLC system (Thermo Fisher Scientific), coupled to an Exploris 480 mass spectrometer (Thermo Fisher Scientific). Peptides were separated by chromatography using a 50 cm column with an inner diameter of 75 µM. The gradient was run from 2% to 40% of ACN in 0.1% FA at a flow rate of 200 nL/minute in 60 min.

Raw data analysis was performed using MaxQuant Software version 2.0.1.0 matching the human proteome (uniprot-proteome_UP000005640.fasta 2022-01-07). Trypsin/P was used to perform database search, with four missed cleavages. Label-Free Quantification was enabled with default values. Carbamidomethylation of cystine residues was considered as a fixed modification. Oxidation of methionines and N-terminal acetylation were considered variable modifications.

MaxQuant proteingroups.txt were further analyzed using Perseus Software version 1.6.15 (Tyanova et al., 2016). 4 and 20 h 5-Aza-2’ treated samples were analyzed separately. Data sets were filtered for potential contaminants or only identified by site. LFQ intensities were Log2 transformed, right-sided Student’s t-test was (FDR 0.05 q-value) performed between His10-SUMO2 enriched samples and their parental control counterparts. All proteins not significantly enriched in at least 3 out of 4 replicates per sample type of His10-SUMO2 samples were removed. Next, two-sided Student’s t-tests (FDR 0.05 q-value) were performed between DMSO and 4 h of 5-Aza-2’ treatment and between DMSO and 20 h of 5-Aza-2’ treatment of the significantly enriched peptides in the SUMO expressing samples. Data for both groups were loaded into VulcanoSer [42] to generate volcano-plots. Hits were considered different when Log2 of LFQ intensities are higher than 1 and statistically significant P of -Log2 1.3.

#### STRING network analysis and Gene ontology

Network analysis of proteins identified by mass spectrometry was performed with the STRING app in Cytoscape version 3.9.1. [43], with a confidence interaction score of 0.4. Sub cluster analysis was performed with the Molecular Complex Detection (MCODE) plug-in (degree cutoff of 2; Node Score cutoff: 0.2: k-Core 2 Max. Depth: 100). Gene ontology analysis was performed with GO consortium web tool (www.geneontology.org). The PANTHER overrepresentation test (released 2022-02-02) was used, GO Ontology database DOI: 10.5281/zenodo.6399963 released 2022-03-22. Proteins were analyzed for overrepresentation in GO molecular function, GO cellular component and GO biological process with Fisher’s exact test and FDR corrected. Only pathways with a fold change <20 were represented.

### Cell viability

Lymphoma cell panel cell lines were seeded in 96-well plate format, cells were seeded at a density of 1 × 10^5^ cells/mL except SUDHL4 and SUDHL5 that were seeded at a density of 5 × 10^5^ cells/mL. Cells were treated for 4 days with increasing concentrations of TAK981 (0.0001–1 µM) or 5-Aza-2’ (0.01–20 µM); 0.01% DMSO was used as control (Fig. 4A, B). For synergy analysis (Fig. 4D, E), a dose range of 5-Aza-2’ (0.01–0.25 µM) with or without 0.025 µM of TAK981 was used. Presto Blue viability reagent (A13261, Merck) was added 1:10 into cell culture medium for 1 h at 37 °C and 5% CO_2_. Fluorescence was measured with a plate reader (Victor X3, Perkin Elmer, Waltham, MA, USA) at 544/591 nm. Three technical replicates were used within each of three biological replicates performed for the viability assays performed. The excess overbliss model [32] was used to calculate the synergistic score, using the following formula with Fa as the fractional activity: Excess overbliss = (Fa1 + 2 − [(Fa1 + Fa2) − (Fa1 × Fa2)]) × 100,

### In vivo tumor model

Animal procedures were performed according to AVD116002017891 appendix 2 which was approved by the Central Committee of animal experiments (CCD, The Hague, The Netherlands) according to the European legislation (EU 2010/63/EU) and animal experimental committee of Leiden University. Male NOD.Cg-Prkdc<scid>IL2rg < tm1Wjl>SzJ (NOD scid gamma, NSG) mice were housed in the animal facility of Leiden University Medical Centre, in separately ventilated cages and fed ad libitum. Mice 7–12 weeks of age were injected intravenously with 1 × 10^5^ Namalwa cells transduced with luciferase (pCDH Luciferase/tdTomato) 100 uL PBS. For tumor imaging, 150 mg/kg D-luciferin potassium salt (Synchem, Elk Grove Village, IL, USA) was injected intra peritoneal, 7 min past the injection, mice were imaged using the IVIS spectrum Xenogen (Perkin Elmer). Treatment with 25 mg/kg TAK981 and/or 2.5 mg/kg 5-Aza-2’ or HPBCD buffer 20%, was started at day 8 post IVIS measurement indicating an average tumor signal of 1 × 10^5^ BLI (photons/s/cm^2^/r) and quantified in photons/s/cm^2^/sr using Living Image 3.0 (Caliper LifeSciences, Waltham, MA, USA).

28 mice were divided over 4 groups (*n* = 7 per group), tumor growth was measured twice weekly followed by intra peritoneal injections with the treatment. Two mice were lost from the experiment upon tumor injection and before treatment and measurement start, resulting in *n* = 6 for Buffer and 5-Aza-2’ treated groups and *n* = 7 for TAK981 and TAK981 + 5-Aza-2’ treated groups. Mice were divided over the groups depending on their BLI signal at day 7 post tumor injection. The average BLI signal for all the groups was the same at start of treatment. No other randomization or blinding of the researcher was implemented. Mice were sacrificed when the BLI level reached 1 × 10^7^ P/s/cm^2^/r or upon meeting humane endpoints. Sample size calculation: two-tailed, alpha = 0.05, Power 0.80; standard deviation (in the control group) = 20%, effect size (difference control and treatment group) = 40%, shows that a group size of 7 animals per condition is sufficient.

Statistical analysis. Graphpad Prims version 9.3.1. was used to calculate significance via Ordinary Two-way ANOVA, followed by Tukey multiple comparisons to calculate differences between every treatment alpha of 0.05 was considered significant. Equal variability of groups is assumed, otherwise Geisser-Greenhouse correction is implemented.

## Supplementary information

Supplement

Dataset Gene Ontology

Data set mass spectrometry

## Data Availability

The mass spectrometry proteomics data have been deposited to the ProteomeXchange Consortium via the PRIDE partner repository with the dataset identifiers PXD038617 and PXD038620.
