# Supplementary material for: Inhibition of SUMOylation enhances DNA hypomethylating drug efficacy to reduce outgrowth of hematopoietic malignancies
Source: Leukemia. 2023 Feb 15;37(4):864–76. doi: 10.1038/s41375-023-01838-8 (PMC10079526; doi:10.1038/s41375-023-01838-8)
Supplement: Supplementary file 1 — Supplement [file 41375_2023_1838_MOESM1_ESM.pdf]

Supplement

Inhibition of SUMOylation enhances DNA hypomethylating drug efficacy to reduce outgrowth of hematopoietic malignancies

Jessie S. Kroonen[1], Ilona J. de Graaf[1], Sumit Kumar[1], Dennis F.G. Remst[2], Anne K. Wouters[2], Mirjam H.M. Heemskerk[2], Alfred C.O. Vertegaal[1#]

[1]*Department of Chemical and Cell Biology, Leiden University Medical Centre, Leiden, The Netherlands*

[2]*Department of Hematology, Leiden University Medical Centre, Leiden, The Netherlands*

#Corresponding author: vertegaal@lumc.nl

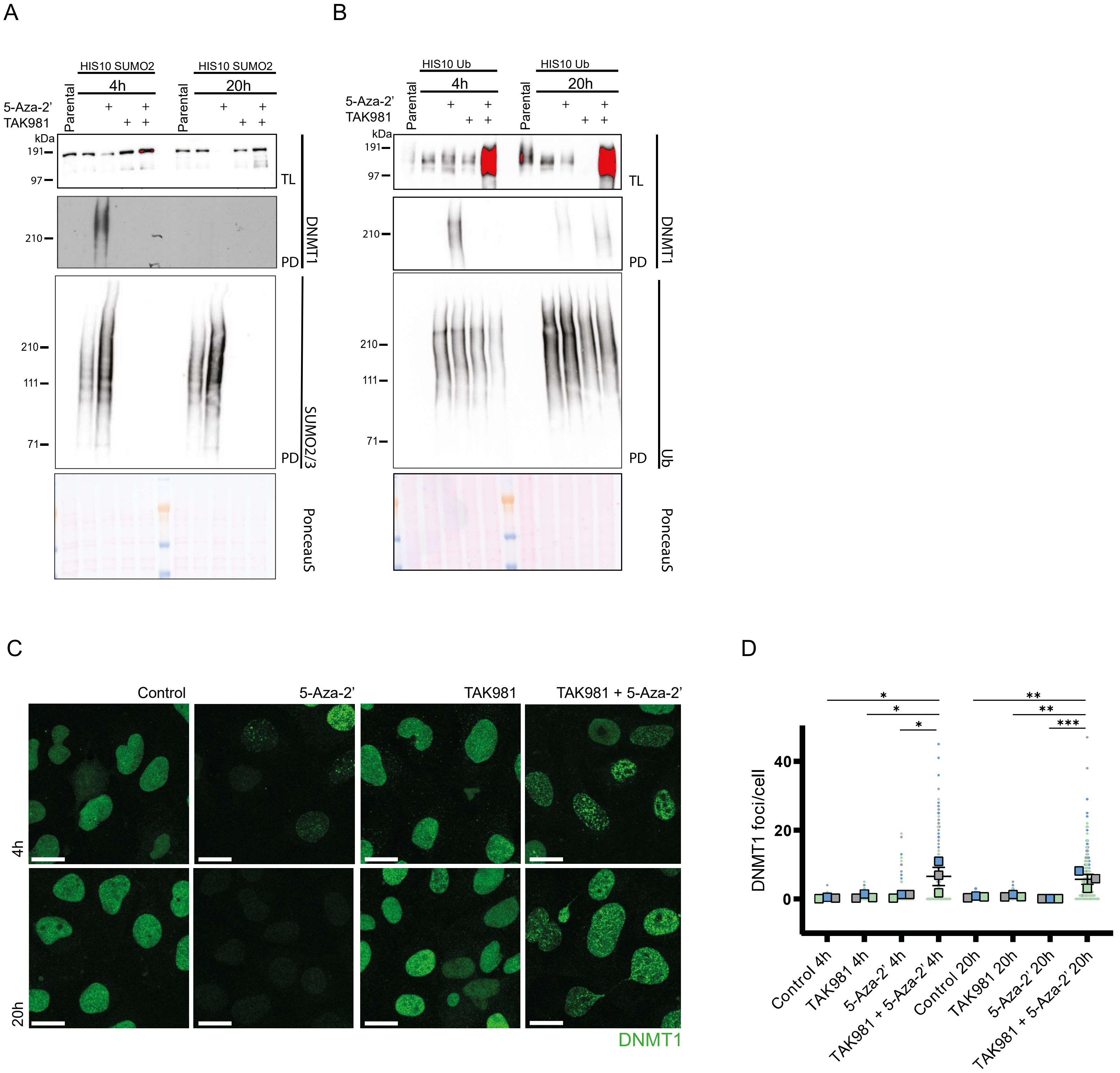
A
B
HIS10 SUMO2
4h
20h
5-Aza-2'
TAK981
Parental
kDa
191
97
210
111
71
TL
PD
DNMT1
SUMO2/3
PonceauS
HIS10 Ub
Ub
C
Control
5-Aza-2'
TAK981
TAK981 + 5-Aza-2'
DNMT1
D
DNMT1 foci/cell
Control 4h
TAK981 4h
5-Aza-2' 4h
TAK981 + 5-Aza-2' 4h
Control 20h
TAK981 20h
5-Aza-2' 20h
TAK981 + 5-Aza-2' 20h

Figure S1

Supplementary Figure 1 SUMOylation inhibition rescues DNMT1 degradation upon 5-Aza-2' treatment and contributes to prolonged presence of DNMT1 in foci in U2OS. **A** & **B** Show Ni-NTA pulldown of His10-SUMO2 and His10-ubiquitin respectively. Parental U2OS cells and U2OS cells stably expressing His10-SUMO2 or His10-ubiquitin were treated for 4 or 20 hour with 1 µM 5-Aza-2' and/or 1 µM TAK981 or DMSO 0.1% as control. Total lysate (TL) and elutions form His10 pulldowns (PD) were analyzed by immunoblotting using antibodies directed against DNMT1, SUMO2/3 or ubiquitin. Equal loading was verified with Ponceau S staining. **C** DNMT1 foci visualized by confocal microscopy. U2OS cells were treated for 4 or 20 hour with 1 µM 5-Aza-2' and/or 1 µM TAK981 or DMSO 0.1% as control on glass coverslips. Representative images are depicted. Scale bar represents 20 µm. **D** Graph depicts DNMT1 foci quantification of images from **C.** Dots represent numbers of DNMT1 foci/cell using ~100 cells per replicate (n=3) * ≤ 0.05, **≤0.01, ***≤0.001.

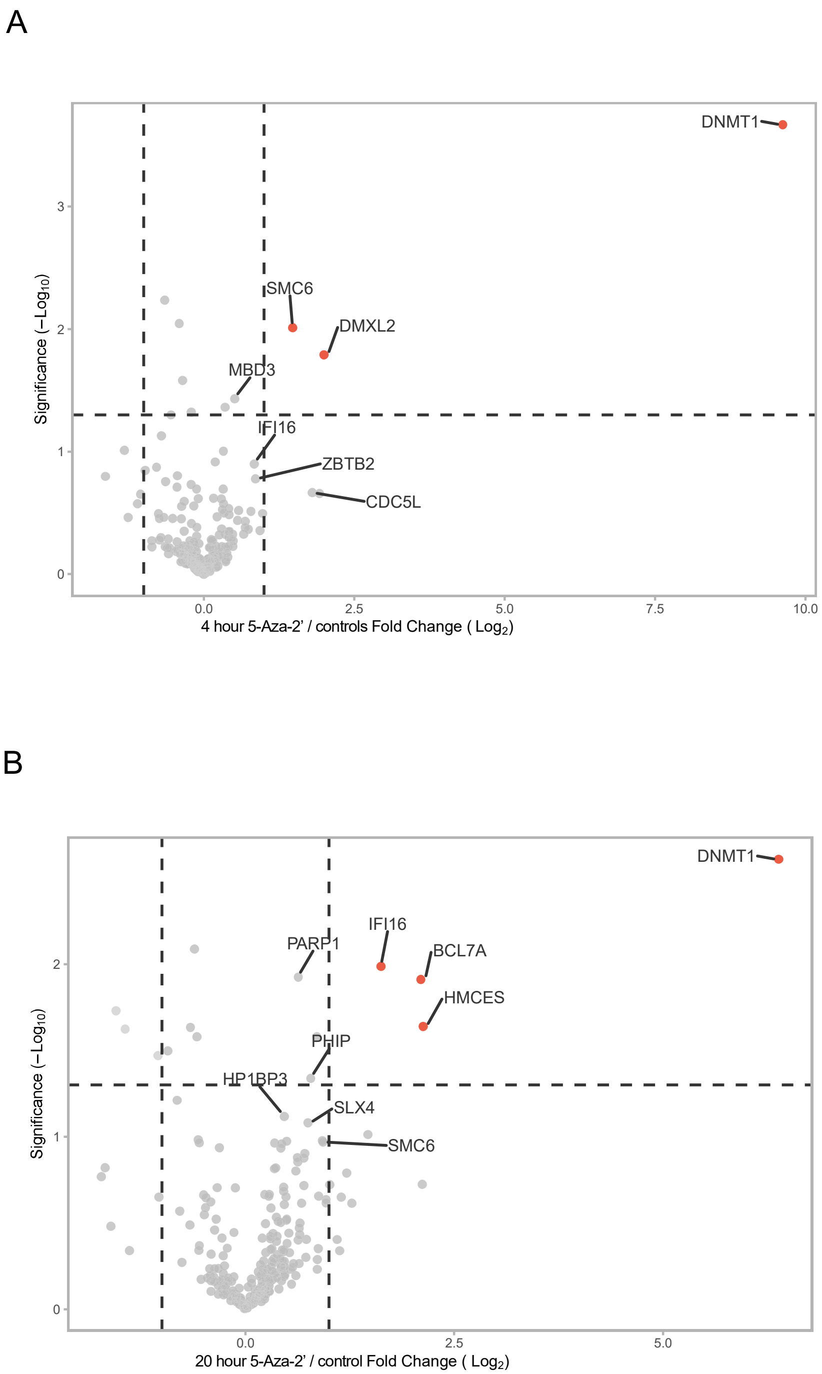
A
DNMT1
SMC6
DMXL2
MBD3
IFI16
ZBTB2
CDC5L
Significance (−Log10)
0
1
2
3
0.0
2.5
5.0
7.5
10.0
4 hour 5-Aza-2' / controls Fold Change (Log2)
B
DNMT1
IFI16
PARP1
BCL7A
HMCES
PHIP
HP1BP3
SLX4
SMC6
Significance (−Log10)
0
1
2
0.0
2.5
5.0
20 hour 5-Aza-2' / control Fold Change (Log2)

Figure S2

Supplementary Figure 2 5-Aza-2' treatment induces SUMOylation of DNA damage response factors in Namalwa cells. **A** & **B** Volcano plots of all identified SUMOylated proteins in Namalwa cells upon 4 and 20 hour of 5-Aza-2' treatment (1 µM) compared to control. His10-SUMO2 conjugated were enriched via Ni-NTA pulldown of His10-SUMO2, followed by trypsin digestion and LFQ mass spectrometry. Peptides were identified by LC-MS/MS. Dashed lines visualize cut off at a foldchange of two (log2=1) and *p-value* of 0.05 (-log10=1.3) (n=4).

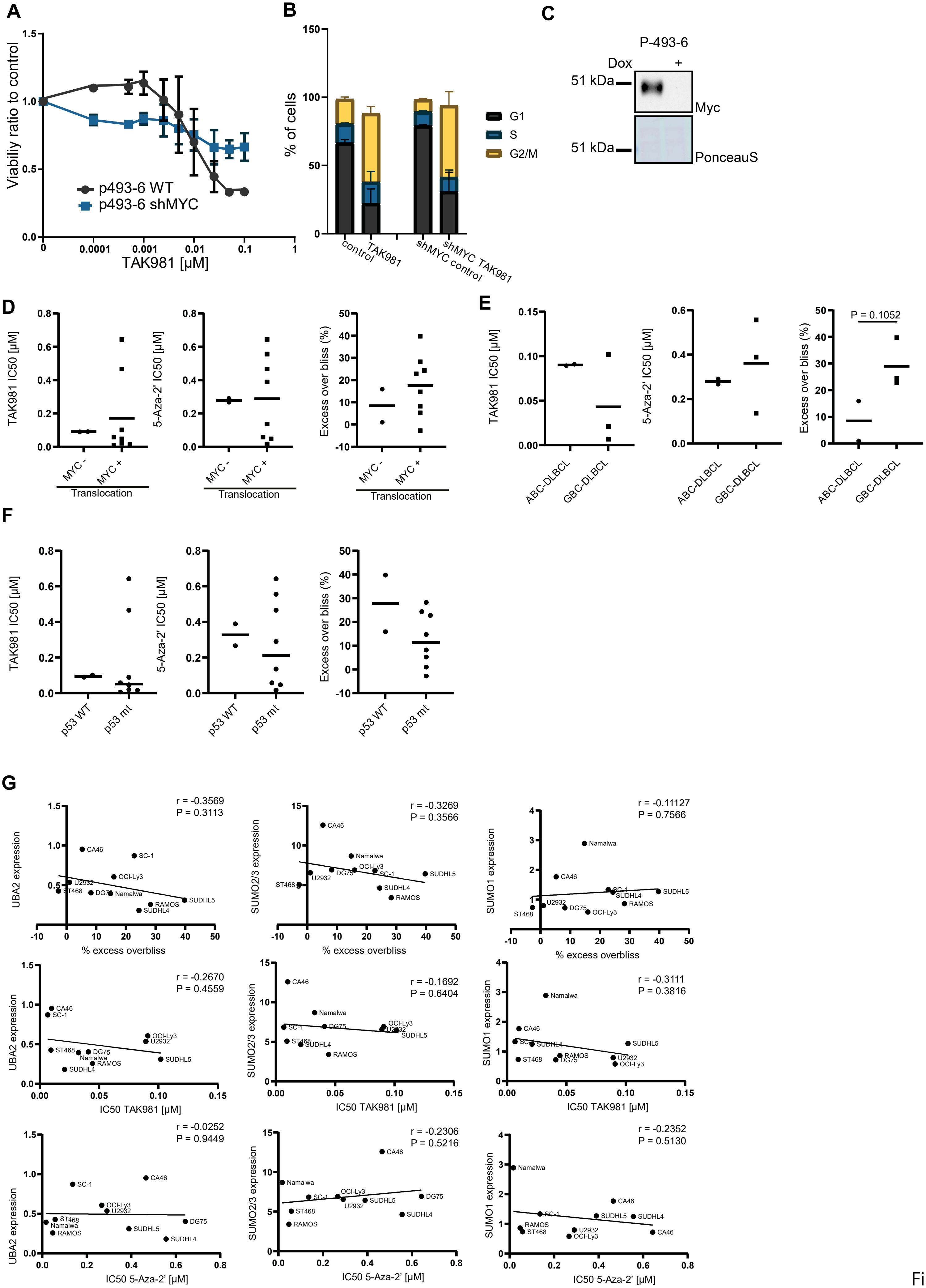
A
Viabiliy ratio to control
TAK981 [µM]
p493-6 WT
p493-6 shMYC
B
% of cells
G1
S
G2/M
control
TAK981
shMYC control
shMYC TAK981
C
P-493-6
Dox
+
51 kDa
Myc
51 kDa
PonceauS
D
TAK981 IC50 [µM]
5-Aza-2' IC50 [µM]
Excess over bliss (%)
MYC -
MYC +
Translocation
E
ABC-DLBCL
GBC-DLBCL
P = 0.1052
F
p53 WT
p53 mt
G
UBA2 expression
SUMO2/3 expression
SUMO1 expression
% excess overbliss
IC50 TAK981 [µM]
IC50 5-Aza-2' [µM]
r = -0.3569
P = 0.3113
r = -0.3269
P = 0.3566
r = -0.11127
P = 0.7566
r = -0.2670
P = 0.4559
r = -0.1692
P = 0.6404
r = -0.3111
P = 0.3816
r = -0.0252
P = 0.9449
r = -0.2306
P = 0.5216
r = -0.2352
P = 0.5130
CA46
SC-1
OCI-Ly3
U2932
ST468
DG75
Namalwa
RAMOS
SUDHL4
SUDHL5

Figure S3

Supplementary Figure 3 No correlation for MYC and p53 protein expression in sensitivity towards 5-Aza-2' and/or TAK981 treatment within the B cell lymphoma panel. **A** Burkitt Lymphoma cell line P493-6, with doxycycline-inducible (0.1 µg/mL) knockdown of MYC was treated with TAK981 at a dose range of 0.1 – 100 nM. Subsequently, cell viability was measured after 4 days with PrestoBlue. Viability is depicted as ratio to control. **B** P493-6 treated with and without 0.1 µg/mL doxycycline for knock down of MYC, was also treated with 250 nM TAK981 for 24h as indicated. DNA was stained with Propidium Iodide and DNA content was measured with flowcytometry. The graph represents % of cells per cell cycle stage. **C** MYC expression levels in P493-6 with and without doxycycline (0. 1 µg/mL) from S3A & B were evaluated via immune blotting. **D** IC50 values for 5-Aza-2' (Figure 4B & C), TAK981 (Figure 4A & C) and excess overbliss percentage (Figure 4D & E) were plotted to compare MYC translocated cell lines versus non-mutated cell lines. Differences were calculated with Two-sided t-tests, performed in Graphpad Prism. **E** Comparison of ABC DLBCL versus GBC DLBCL for sensitivity towards IC50 values for 5-Aza-2' (Figure 4B & C), TAK981 (Figure 4A & C) and excess overbliss percentage (Figure 4D & E). Differences were calculated with Two-sided t-tests performed in Graphpad Prism. **F** IC50 values for 5-Aza-2' (Figure 4B & C), TAK981 (Figure 4A & C) and excess overbliss percentage (Figure 4D & E) were plotted to compare sensitivity related to p53 mutational status. Differences were calculated with Two-sided t-tests, performed in Graphpad Prism. **G** Correlation plots of ten lymphoma cell lines and their IC50 values for either TAK981 dose response (Figure 4A & C), 5-Aza-2' dose response (Figure 4B & C) or excess overbliss (Figure 4D & E) versus protein expression quantified from Figure 4F. Linear regression analysis was performed.

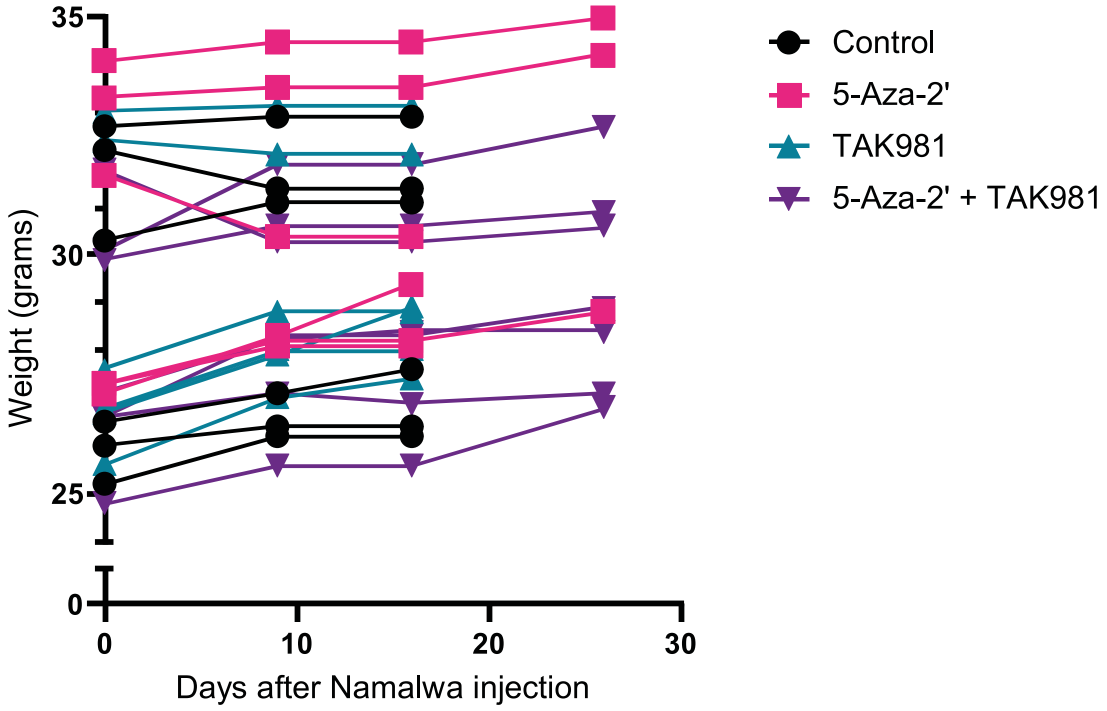

Supplementary Figure 4

Supplementary Figure 4 Mouse weight does not change upon treatment with 5-Aza-2' and/or TAK981. Mouse weight in grams for data presented in Figure 5. Mice were weighed on day 0 of tumor injection and on days 8, 16 and 26 post tumor injection. Mice were treated with compounds on day 8 post tumor injection, followed by bi-weekly injections. All mice were plotted individually.
